# Therapeutic Use of Silver Nanoparticles in the Prevention and Arrest of Dental Caries

**DOI:** 10.1155/2020/8882930

**Published:** 2020-08-12

**Authors:** Claudia Butrón Téllez Girón, Juan F. Hernández Sierra, Idania DeAlba‐Montero, María de los A. Urbano Peña, Facundo Ruiz

**Affiliations:** ^1^Facultad de Estomatología, Universidad Autónoma de San Luis Potosí, C.P. 78000 San Luis Potosí, Mexico; ^2^Facultad de Medicina, Universidad Autónoma de San Luis Potosí, C.P. 78000 San Luis Potosí, Mexico; ^3^Facultad de Ciencias, Universidad Autónoma de San Luis Potosí, C.P. 78000 San Luis Potosí, Mexico

## Abstract

Dental caries is one of the major diseases of the oral cavity affecting humans worldwide. Different alternatives have been used for its control, but its incidence and prevalence are still high. On the other hand, silver has been used for centuries due to its antimicrobial properties. With advances in nanotechnology, the use and research in nanomaterials has increased, recently, and silver nanoparticles have become an essential part of the dental practice, giving materials physical and chemical improvements in their properties, used for their antibacterial capacity preventing and arresting dental caries. The objective of this review was to examine the use of silver nanoparticles, in the treatment of dental caries in the remineralization of teeth hard tissues, as well as the antimicrobial potential, cytotoxicity, and long-term effectiveness.

## 1. Introduction

Dental caries is a disease caused by a specific biofilm that forms acid [1], which begins with demineralization on the enamel surface showing what is called white spot lesion, this stage is represented by white and opaque colour [2, 3], and the main pathogens responsible for dental caries *Streptococcus mutans* and *Lactobacillus* [3–7]. Although, in recent decades, there has been an advance in dental care, dental caries is a global health problem [2, 8] and costly, compromising the health and quality of life of children and adults [1]. The most successful treatments used for the prevention and control of early-stage dental caries are based on the use of fluorides [9–12]. In dentistry, silver has been used for over a century as an antimicrobial agent due to its broad spectrum, low toxicity, and absence of cross-spectrum bacterial resistance [13–18]. Silver nitrate was used for reducing the incidence of caries in the deciduous dentition, as a caries preventive agent for permanent molars, a cavity sterilizing agent, and as a dentine desensitizer [9, 19]. Subsequently, in the 1960s, it was suggested to combine silver with fluoride (silver diamine fluoride) as an anticaries agent for a possible combined effect [20, 21]. However, its clinical application has been limited due to the black pigmentation produced by its application [9, 22–26].

The use of nanotechnology in dentistry has attracted significant attention in the recent years [1], showing novel methods in the prevention and treatment of caries, controlling plaque-related biofilms, and remineralization of primary dental caries [27], among these methods are the formulations of silver nanoparticles (AgNPs) with antimicrobial properties against a wide variety of microorganisms [1, 9, 28–34].

Silver nanoparticles are considered potent antimicrobial agents and have been proven to be effective in vitro against cariogenic bacteria such as *Streptococcus mutans* [35], having an antibacterial activity 25 times greater than chlorhexidine, as well as an antiviral and antifungal activity [33, 36], so multiple studies have proposed their use in various preparations, showing good results in the treatment of early dental caries [4, 37, 38].

For these reasons, the objective of this report is to present a review of the literature on the possible therapeutic use of silver nanoparticles in dentistry, in the remineralization of teeth hard tissues, their mode of action, and biocompatibility.

## 2. AgNPs Formulations in Caries Prevention and Arrest

Dental caries is one of the most widespread diseases in the world, affecting 95% of the population [39, 40] at different stages of their lives [41, 42]. Several studies have designed formulations containing silver nanoparticles against cariogenic pathogens mainly against *Streptococcus mutans*, evaluating their minimum inhibitory concentration (MIC) and minimum bactericidal concentration (MBC) and in the control of biofilm formation; showing that they are good oral antimicrobial agents [28–30, 43], especially at dimensions between 80100 nm, since cytotoxicity increased to dimensions lower than 20 nm [44] has been demonstrated (Table 1).

New formulations have been evaluated against cariogenic pathogens and their cytotoxic activity through haemolytic activity in different concentrations provisionally called silver nanofluoride (NSF). MIC and MBC were, respectively, around 33.54 *µ*g/ml and 50.32 *µ*g/ml. Silver diamine fluoride (SDF) showed similar values. NFS proved to be an antimicrobial agent similar to silver diamine fluoride, but less cytotoxic; however, the effect was not tested or if it stained the enamel surface [37].

Another important aspect to evaluate is the microhardness of the enamel after remineralization with formulations containing AgNPs and evaluating the cariostatic effects using microhardness and microbiological tests as outcome variables, using Saforide®, Cariestop®, Ancarie®, and Ag-Nano (experimental cariostatic agent). Naturally exfoliated deciduous teeth were submitted to an initial test (after a pH cycle to obtain an initial like-caries lesion) and a final microhardness test (after application of the cariostatic agents), with an antimicrobial effect evaluated with *Streptococcus mutans*, *Escherichia coli*, and *Enterococcus faecalis* strains. All the cariostatic showed an improvement in the remineralization of the enamel, in which Saforide® had a higher percentage of microhardness 28.55 ± 11.75 than that of Ag-Nano 14.63 ± 13.38. Also, in the agar diffusion tests, a greater inhibition of *S. mutans*, *Enterococcus faecalis*, and *Escherichia coli* by Saforide® was observed than by Ancarie® and Ag-Nano. However, the MIC Ag-Nano inhibited the growth of microorganisms at a concentration lower than the other agents, remineralizing also the enamel of deciduous teeth with initial like-caries lesion [45].

In another study were prepared dentin discs from previously demineralized premolars. SDF (38%), nano-silver fluoride (NSF) (3.16%, 3.66%, and 4.16%), and propolis fluoride (PPF) (3%, 6%, and 10%) were applied and subjected to pH cycles using a demineralization solution (pH 4.4) for 30 minutes and a remineralization solution (pH 7) for 10 minutes, and this cycle was performed 6 times a day for 8 days. The amount of calcium, phosphate and fluoride ions on the dentine discs surface was compared by energy dispersive X-ray spectroscopy. The results showed that the levels of calcium ions, phosphate, and fluoride in the NSF and PPF groups increased significantly compared to SDF [48]. According to the previous results and with the same procedures, fluorapatite crystals were found on the dentine surface in the SDF and NSF, as well as greater hardness and increased intensity and quality of apatite crystal compounds [49]; on the other hand, the use of optical coherence tomography images showed a greater remineralization effect of NSF compared to sodium fluoride (NaF) in deciduous teeth [50].

## 3. Clinical Evidence

Although most studies on cariogenic bacteria are still *in vitro*, in recent years, controlled clinical trials have been conducted to measure the efficacy of silver nanoparticles as anticariogenic agents in remineralization.

A controlled clinical trial was performed on decayed teeth *in vivo.* A formulation of nanoparticle silver, fluoride, and chitosan (NSF) was used as the experimental group and water as the control group. The caries was diagnosed clinically, and only one application was made, with an evaluation of seven days, five and twelve months. On the seventh day, 81% of the teeth in the treatment group had arrested cavities and 0% in the control group. After five months, the treatment group had 72.7% with arrested caries, and the control group had 27.4%. At 12 months, 66.7% of the caries lesions treated with NSF were still arrested, while the control group had 34.7%. No dark stains were observed on the teeth [4].

In a similar study, the effect of AgNPs added to a commercial fluoride varnish on remineralization of deciduous teeth was evaluated. The study was conducted in children with white spot lesions in anterior deciduous teeth diagnosed with the DIAGNOdent® laser cavity detection pen to measure tooth demineralization. A mixture of silver nanoparticles (powder) and fluoride varnish (Fluor Protector Varnish®, Vivadent, Schaan, Liechtenstein) was prepared at 0.1% wt. The teeth were assigned, one tooth of each hemi arc to the experimental treatment which was fluoride varnish adding 0.1% AgNPs and the other for the control treatment, which was commercial fluoride varnish (Fluor Protector Varnish®). Each tooth was submitted to these treatments once a week for 3 weeks, and measurements were taken again with the DIAGNOdent® at three months to evaluate changes in remineralization, being higher in treatment with AgNPs with a *p*=0.001 [46].

In another research, the efficacy of silver nanoparticles added to a pit and fissure sealant was evaluated in permanent molars to determine the demineralization of this mixture against a control group. They observed that the conventional sealant presented an average microleakage of 30.6%, and the sealant added with silver nanoparticles showed 33.6% (*P* = NS). A three times greater reduction in fluorescence was found in the AgNPs group compared to the conventional group (*p* <  0.05). No associations were found based on sex or age, concluding that the sealant with silver nanoparticles significantly reduced dental demineralization and probably increased remineralization compared to the conventional sealant [47].

Finally, a study was recently conducted using nanosilver 5% incorporated sodium fluoride (NSSF) dental varnish and 38% SDF. No significant differences were observed between the NSSF and SDF groups during their 12-month follow-up. NSSF did not cause dark staining of dentinal tissue [51].

## 4. Discussion and Perspectives

SDF has been used as a gold standard in several investigations [23–25, 52–59] in different concentrations in deciduous teeth with a high risk of active caries [20, 59, 60], effectively preventing and controlling them [23, 24, 57, 61–63]. However, it has shown adverse effects, such as causing ulcerations and staining in oral mucosa [64] that are painful due to accidental contact with the solution [20, 65], which may disappear within 48 hours [21, 65] and the black staining of carious tissue [65–67] due to the oxidation process of the ionic silver contained in its formulation [4, 68]; however, as previously demonstrated, the use of a cariostatic agent with silver nanoparticles does not cause darkening in demineralized teeth [46–48, 51]. Besides, practically all studies have demonstrated the anticariogenic and remineralizing effect of silver nanoparticles alone [45] and in combination with various components, being superior to conventional treatments [48–51] such as silver diamine fluoride when applied once a year, with the advantage of not staining black dental tissue, the reason being they do not form oxides when in contact with oxygen in the environment, no metallic taste, and lower cost compared to SDF [4, 51].

Although the cariostatic effect on dental tissues has not been clarified yet [4], the effectiveness in arresting cavities can be explained by the synergism of the components of their formulation (nanoparticles of silver, chitosan, and fluoride) [69]. Chitosan is a biocompatible, biodegradable, and nontoxic biopolymer widely known for its activity against a wide range of microorganisms [69, 70], including *Streptococcus mutans* [69] and *Streptococcus sanguinis* [71], and by adding the antibacterial mechanism of silver nanoparticles, the silver ions can adhere to the cell wall and cytoplasmic membrane due to electrostatic attraction and affinity to sulfur proteins leading to disruption of the bacterial envelope. Once the ions enter the cells, the respiratory enzymes are deactivated, generating reactive oxygen species. As phosphorus and sulphur are components of DNA, the interaction of silver ions with these elements can cause problems in DNA replication and cell reproduction. Furthermore, silver ions can inhibit protein synthesis causing structural changes and cell death [72]. Besides, the reduction in the size of silver nanoparticles implies an increase in the contact surface, which is an important condition for the antimicrobial effects of silver and which could prevent black stains on teeth [36], as it occurs after the application of SDF [20] and reduces toxicity [36]. Therefore, the activity and stability of silver nanoparticles are believed to be influenced by the nature of their stabilizing agent (chitosan) within their formulation, having a low level of constant interaction between silver nanoparticles and bacteria [35, 73].

Although the use of silver nanoparticles in the medical and dental field is of great importance, knowledge about the effect of human exposure and the possible toxicity of these products is limited [74]. However, haemolytic activity tests were performed to evaluate the cytotoxicity of NSF compared to SDF and found not to cause damage to the human erythrocyte membrane, regardless of the blood type. When evaluating absolute cytotoxicity values, NSF is less toxic than SDF (*p*=0.05) [37].

The present review showed that these formulations can prevent and arrest demineralization of deciduous tooth enamel, so it is important to take into account that these have a thickness of their enamel layer thinner than the permanent teeth [75–77], being more prone to the caries progression in comparison with the permanent tooth [76–78]. Despite these disadvantages, the deciduous tooth is more sensitive to fluoride treatments, because its enamel has a higher permeability which is approximately 150 times greater than that of a permanent tooth [35, 75]. Since this enamel is more sensitive to acid action, the bactericidal potential of silver will be able to increase the effect of fluoride while preserving the remineralization potential [50].

Currently, the use of these formulations with silver nanoparticles and fluoride in different concentrations display a positive effect on dental hard tissue for enamel remineralization in deciduous and permanent teeth, increasing the remineralizing, and they may enhance the performance of fluoride with antimicrobial action of silver added to these formulations, preventing and arresting the carious lesion without causing staining. However, no publication has reported the long-term stability and antibacterial effect of silver nanoparticles and fluoride.

## 5. Conclusions

The investigations conducted in laboratory and clinical trials have shown the superiority of silver nanoparticle compounds in the prevention and arrest of dental caries, without the adverse effect of dental pigmentation. This therapy is easy to use and of a lower cost than SDF.

The benefit of the combination of AgNPs with other components must be examined to know its interaction with dental tissues, as well as to determine ideal concentrations, the optimal size of AgNPs, delivery vehicles, doses related to cellular toxicity, and adverse effects. These studies can guide future clinical application protocol for permanent and deciduous dentition whose structure is different, and the behaviour of the formulations added with silver nanoparticles could show different results.

## Figures and Tables

**Table 1:** Use of silver nanoparticles in the prevention and arrest of the dental caries.

Author/year	Type of study and intended effect on caries	Assessment method/combined strateg**y**	Particle size (nm) and shape/method of obtaining	Outcome
Targino et al. [37] 2014	Experimental *in vitro* caries prevention	UV-visible, TEM, MIC 399.33 *µ*g/ml AgNPs, 2,334 *µ*g/mL chitosan, and 10,147 *µ*g/ml NaF	5.9 ± 3.8, spherical chemical synthesis	NSF is a promising anticaries agent, with low toxicity and the potential advantage of not to stain teeth black
Scarpelli et al. [45] 2017	Experimental *in vitro* caries prevention	Microhardness testing (knoop-type penetrator (KHN)) superficial and internal, ADT MIC 0.016% AgNO_3_, and sodium citrate	NA NA green synthesis	Ag-nano remineralized deciduous dental enamel and better bactericidal activity; it does not cause darkening in demineralized teeth
Santos et al. [4] 2014	Controlled clinical trial caries arresting	ICDAS II 376.5 *µ*g/ml AgNPs, 28,585 *µ*g/ml chitosan, and 5028.3 *µ*g/ml NaF	3.2 ± 1.2, spherical chemical synthesis	NFS is effective to arrest active dentine caries in deciduous teeth and not to stain teeth with one application per year
Butrón-Téllez Girón et al. [46] 2017	Controlled clinical trial caries prevention	UV-visible, TEM DIAGNOdent® AgNPs (powder), and Fluor Protector Varnish® at 0.1 %wt	46 pseudospherical chemical synthesis	Fluoride varnish with AgNPs was better in the dental remineralization than conventional varnish
Salas-López et al. [47] 2017	Controlled clinical trial caries prevention	DIAGNOdent® AgNPs 98 *µ*g/ml and Clinpro^TM^ pit, an fissure sealant	40–80 NA chemical synthesis	AgNPs added sealant increased remineralization in permanent first molars compared to the conventional sealant
Soekanto et al. [48] 2017	Experimental *in vitro* caries prevention	EDX 3.16, 3.66 y 4.16% AgNO_3_, 5 ml gelatin, glucose 13.3 g/40 ml, distilled water, and 4.4 g NH_4_F	NA NA green synthesis	NSF^*∗*^ showed an increase in calcium, phosphate, and fluoride ion levels compared to SDF
Soekanto et al. [49] 2017	Experimental *in vitro* caries prevention	SEM XRD 3.16, 3.66 y 4.16% AgNO_3_, 5 ml gelatin, glucose 13.3 g/40 ml, distilled water, and 4.4 g NH_4_F	NA NA green synthesis	NSF^*∗*^ increases the intensity and quality of apatite crystal components, increasing remineralization
e Silva et al. [50] 2019	Experimental *in vitro* caries prevention	OCT .3 g/60 ml chitosan, 0.012 mol/L AgNO_3_, and 5500 ppm NaF	8.7 ± 3.1 NA chemical synthesis	NSF has the best effect against caries compared to conventional fluoride treatments
Tirupathi et al. [51] 2019	Controlled clinical trial caries arresting	UNC-5 probe AgNPs power 0.5 grams containing polyvinyl pyridoline added to 10 ml of 22,6000 ppm of sodium fluoride varnish (FLUORITOP^TM^-SR)	−100 NA NA	Annual application of NSSF is better than or equal to SDF in preventing the progression of dentinal caries in deciduous molars, and it does not cause dark staining of dentinal tissue

AgNO_3_: silver nitrate, AgNPs: silver nanoparticles, TEM: transmission electron microscopy, OCT: optical coherence tomography, NaF: sodium ﬂuoride, MIC: minimum inhibitory concentration, NSF: silver nitrate, chitosan, and fluoride, NH_4_F: ammonium fluoride, ICDAS II: International Caries Detection and Assessment System, EDX: energy-dispersive X-ray spectroscopy, NFS^*∗*^: silver nitrate: gelatin, glucose, and ammonium fluoride, NSSF: nanosilver 5% and sodium fluoride, ADT: agar diffusion test, EDX: energy-dispersive X-ray spectroscopy, SEM: scanning electron micrograph, XRD: X-ray diffraction.

## Data Availability

The data that support the findings of this study are available on request from the corresponding author.
